# Bilberries potentially alleviate stress-related retinal gene expression induced by a high-fat diet in mice

**Published:** 2012-09-07

**Authors:** Otto T. Mykkänen, Giedrius Kalesnykas, Michiel Adriaens, Chris T. Evelo, Riitta Törrönen, Kai Kaarniranta

**Affiliations:** 1 Institute of Public Health and Clinical Nutrition, Department of Clinical Nutrition, Food and Health Research Centre, University of Eastern Finland, Kuopio, Finland; 2 Department of Bioinformatics-BiGCaT, Maastricht University, Maastricht, The Netherlands; 3 Department of Ophthalmology, University of Eastern Finland, Kuopio, Finland; 4 Department of Ophthalmology, Kuopio University Hospital, Kuopio, Finland

## Abstract

**Purpose:**

Obesity- and diabetes-associated visual impairment and vascular dysfunctions are increasing as causes of vision loss. The detailed mechanisms of how obesity and diabetes affect eye health are still largely unknown, but animal models have been useful in exploring the effects of potential protective compounds, i.e., compounds characterized by antioxidant and anti-inflammatory properties. These properties occur in anthocyanins, and bilberries (European wild blueberries, *Vaccinium myrtillus*) are a major source of dietary anthocyanins in Nordic diets. The main aim of the present work was to study the protective effects of dietary bilberries (BB) on the level of gene expression in retinas in mice that develop obesity when fed a high-fat diet (HFD).

**Methods:**

Mice (n=6 per group, four groups) were fed ad libitum a normal control diet (NCD), a HFD, or a diet with 5% bilberries (NCD+BB, HFD+BB) for 12 weeks. Food consumption, weight gain, and blood pressure were measured during the feeding period and whole blood serum markers of obesity at sacrifice. Retinas were collected, and RNA extracted from all 24 mice and pooled samples from four mice per group were hybridized to Mouse-Ref8 V2 Expression BeadChips (Illumina platform) with 25,697 probes for genes and transcript variants. The expression profiles in the retinas were analyzed using R, PathVisio, and DAVID to screen for high fat–induced changes as well as for bilberry-induced changes in the HFD up- or downregulated transcripts.

**Results:**

The HFD and HFD+BB groups gained weight from week 5 and final weight, blood glucose, serum free fatty acids, and systolic blood pressure as compared to mice fed the control diets (Mann–Whitney’s U-test, p<0.05). Bilberries had no significant effect on these parameters other than a trend to reduce systolic blood pressure in the HFD-fed mice (101±4 versus 113±9 mmHg, p=0.10). Gene ontology enrichment analysis of 810 differentially expressed genes (F-test, p<0.05) in the retina displayed differential regulation of genes in ontology groups, mainly pathways for apoptosis, inflammation, and oxidative stress, especially systemic lupus erythematosus, mitogen-activated protein kinase, and glutathione metabolism. Mice fed a HFD had increased retinal gene expression of several crystallins, while the HFD+BB mice showed potential downregulation of these crystallins when compared to the HFD mice. Bilberries also reduced the expression of genes in the mitogen-activated protein kinase (MAPK) pathway and increased those in the glutathione metabolism pathway.

**Conclusions:**

HFD feeding induces differential expression of several stress-related genes in the mouse retina. Despite minor effects in the phenotype, a diet rich in bilberries mitigates the upregulation of crystallins otherwise induced by HFD. Thus, the early stages of obesity-associated and stress-related gene expression changes in the retina may be prevented with bilberries in the diet.

## Background

High-fat diet (HFD) mouse models are invaluable tools for clarifying the development of obesity at the tissue level. The C57BL (black) strain is the most widely used inbred strain highly susceptible to HFD-induced obesity and hyperglycemia, and serves as a background strain for numerous transgenic mouse models [1–3]. When nearly half of the energy in the mice’s diet is derived mainly from saturated fat, these mice readily develop obesity, insulin resistance, and systemic inflammation. Prolonged HFD and its modifications lead to increased systolic blood pressure and impaired lipid and glucose homeostasis, and this model has been widely used to study type 2 diabetes (T2D) [3–5]. Obesity has been linked to several ocular diseases, such as diabetic retinopathy, microvascular complications, age-related macular degeneration (AMD), and diabetic cataracts [6], but the associations remain unclear and sometimes inconsistent. HFDs increase features associated with dry AMD in aged mice or when consumed for long periods [7], but short-term changes in mice have not been fully explored. Furthermore, HFDs are generally used in combination with other challenges to mimic features of dry AMD, whereas overexpression of vascular endothelial growth factor or laser treatment have been used to create choroidal neovascularizations and thus to develop models of wet AMD [8]. Retinal gene expression studies have identified several genes involved in inflammation, the antivascular barrier, and neurodegeneration with increased apoptosis and vascular permeability with streptozotocin (STZ) induced diabetic rats [9–11]. In addition, genetic Ins2^Akita^- or STZ-induced diabetic mice also have neurovascular and inflammatory genes associated in the progression of diabetic retinopathy [12,13]. Therefore, inflammatory components appear to play a crucial role in the development of obesity-associated ocular diseases, but as far as we are aware, no studies on retinal gene expression patterns after an HFD have been published previously.

Bilberries are rich in polyphenols, especially anthocyanins, which belong to the flavonoids [14,15]. Bilberries are generally recognized as low energy foods, containing little or no saturated fat, cholesterol, and sodium, and as a good source of dietary fiber and antioxidant vitamins E and C. The anthocyanins are the most abundant flavonoids present in bilberries and have been shown to be potential antioxidants [16]. These compounds can penetrate the nervous system even after short-term feeding [17], and they have been reported to be able to reduce age-associated oxidative stress and the related cognitive decline [18]. Furthermore, anthocyanins displayed the potential to ameliorate cardiovascular diseases [19] and obesity-related pathological changes in rodent models [20–23], but findings using whole bilberries as anthocyanins sources have not demonstrated similar efficacy [24–26]. Due to their antioxidative and anti-inflammatory properties [27,28], anthocyanins and their extracts may have potential benefits in eye health [29–31], neural functions [32] and age-related retinal stress [33].

In this study, we used microarrays combined with a well characterized mouse model to screen for the genes expressed in the retina that could potentially explain the effects of bilberries in the diet at the early stage of HFD-induced obesity. As far as we are aware, this is the first study using 45% kcal from an HFD and mice to study the short-term effects on retinal gene expression.

## Methods

### Animals and study design

Mice (male C57BL/6J, n=24) 6 weeks of age were obtained from the animal center of the University of Eastern Finland and randomly divided into four groups (six mice per group) and housed in pairs. They were kept in a constant environment at 21 °C with an automated 12 h:12 h light-dark cycle: 7:00–19:00 day (light), 19:00–7:00 night (dark). The mice were fed ad libitum for 12 weeks with a normal control diet (NCD; 10% kcal fat), high-fat diet (HFD; 45% kcal fat), or these diets supplemented with 5% (w/w) of freeze-dried bilberries (BB), and had free access to tap water. The fat content in the HFDs was increased by the addition of lard, a rich source of saturated fat. The berry diets were prepared by the manufacturer (Research Diet, Inc., New Brunswick, NJ) to match the respective controls in terms of the carbohydrate (sucrose and cellulose) and potassium (potassium citrate) content. The Finnish national food composition database (Fineli®) was used for estimating the nutrient content of bilberries. The energy nutrient contents of the diets are shown in Table 1 and in detail as provided by the manufacturer in Appendix 1. The anthocyanin contents of the freeze-dried berry powder and the BB diets were analyzed with high-performance liquid chromatography as previously described [34] and expressed as mg/g diet as anthocyanidin (aglycon) equivalents. Feed and water consumption of each cage were monitored for three 5-day periods (weeks 2, 6, and 12), and the average feed intake was used to calculate the anthocyanins and energy intake.

**Table 1:** Nutrient contents of the normal control diet (NCD) and high-fat diet (HFD) with or without 5% bilberries (BB).

	**Control diets**				**Bilberry diets**			
	**NCD**		**HFD**		**NCD+BB**		**HFD+BB**	
**Diet**	**g**	**% kcal**	**g**	**% kcal**	**g**	**% kcal**	**g**	**% kcal**
Proteins	19.2	20	23.7	20	18.7	20	23.0	20
Carbohydrates	67.3	70	41.4	35	64.0	70	38.8	35
Fat	4.3	10	23.6	45	4.2	10	23.0	45
Total	90.8	100	100	86.9	84.9	100		
kcal/g	3.8		4.7		3.8		4.7	

The NCD and HFD with BB contained 0.92 and 0.83 mg/g anthocyanins, respectively.

Weight gain was measured weekly and blood pressure during the last two weeks of the study (weeks 11–12). The systolic blood pressure and heart rate were measured with a non-invasive tail cuff method with slight modifications as described previously [35]. Briefly, the mice were acclimatized individually to the measurement conditions (restrainers and warming) for 2–3 days and trained for 5 days with sham measurements before data were collected from 10 repeated cuff measurements in prewarmed restrainers at 32 °C at the same time of day using an automated inflation system (IITC Inc. Life Sciences, Los Angeles, CA). Data collected from at least two measurements with <15% coefficient of variation of the mean were used to obtain an average systolic blood pressure (mmHg) and heart rate (HR, pulse per minute) for each mouse.

The mice were euthanized at the end of the feeding trial following 1–2 h fasting using CO_2_ gas asphyxiation until loss of vital signs before cervical dislocation. Both retinas were removed under a light microscope and found to be free of pigmentation (retinal pigment epithelium [RPE] cells). Samples were snap frozen in liquid nitrogen before RNA was extracted. Whole blood glucose was analyzed with the One Touch Ultra glucose meter and strips (Life Scan, Milpitas, CA). Serum samples were collected in Microvette CB 300 capillary tubes (Sarsted, Nümbrecht, Germany) after 3 min centrifugation at 12 000× g to determine free fatty acids (FFAs) with a standard enzymatic protocol with Konelab 20XTi analyzer (Thermo Electron Corp., Vantaa, Finland). All blood and tissue samples were snap frozen in liquid nitrogen and aliquots stored at −70 °C.

The study protocol was approved by the Finnish National Animal Ethics Committee in the State Provincial Office of Southern Finland, adherent to the European Communities Council Directive (86/609/EEC) and comparable to the guidelines published by the Institute for Laboratory Animal Research.

### RNA extraction and microarray transcriptomics

Total RNA was extracted from both retinas of four mice per group using QIAzol and cleaned up with the RNeasy Lipid Tissue Mini kit (Qiagen, Valencia, CA). The biotinylated cRNA was pooled. Isolated RNA and cRNA concentrations and quality were checked with Nanodrop (ND-1000, Wilmington, DE). The quality was controlled with BioRad’s Experion electrophoresis station at the Finnish Microarray and Sequencing Centre (FMSC, Turku, Finland) before hybridizations. The raw and normalized data are available at the National Center for Biotechnology Information (NCBI) Gene Expression Omnibus (GEO) with the series accession number GSE34154.

In brief, the RNA was isolated from homogenates in 1 ml of QIAzol lysis reagent, and RNA extracted according to the manufacturer’s protocol (RNeasy Lipid Tissue Handbook) with the additional Dnase step (Qiagen’s Rnase-Free DNase Set). The Dnase-treated RNA was eluted in 50 μl of water and amplified and biotinylated overnight (15 h) with Ambion's Illumina TotalPrep RNA Amplification kit (Austin, TX). Pooled cRNA 750 ng from four samples (187.5 ng) per group was hybridized at 58 °C overnight (19 h) to each array using the standard Illumina protocol with 1 μg/ml streptavidin-Cy3 detection (Amersham, Piscataway, NJ). The Mouse-Ref^8^ V2 R0 Expression BeadChips (Illumina Inc., San Diego, CA; Platform GPL6885) were scanned with the Illumina Bead Array Reader and results extracted with BeadStudio v3.3.7 (Illumina Inc.).

### Illumina bead array data and gene set enrichment analysis

Bead-level data were extracted from the bead array scans and summarized using Illumina BeadStudio software without any normalization or background correction. The resulting gene-specific bead-summary data were used as the basis for the gene expression analyses in the statistical programming language R, primarily using beadarray [36] and limma [37] packages from the Bioconductor project [38].

For each group, we used the control probe signal intensities (Biotin, Cy3 hybridization efficiency, housekeeping genes, labeling efficiency, low stringency hybridization efficiency, and negative controls), box-and-whisker plots of all log_2_ expression values, M versus A plots of all possible combination of arrays, and hierarchical clustering at the array level to check for batch effects and other technical biases. After quality control, the bead summary data were reannotated using the most recently available annotation, and normalized using quantile normalization.

Gene set enrichment analysis (GSEA) was performed with significantly differently expressed genes (p<0.05, n=810), using the functional annotation tools available from the Database for Annotation, Visualization and Integrated Discovery (DAVID). Pathway analysis on the same set of genes with various approaches was performed in PathVisio and DAVID.

We used the DAVID functional annotation tools [39] to obtain a global overview of the biologic processes that have been regulated by the diets. First, enrichment for all Kyoto Encyclopedia Gene and Genomes (KEGG) pathways was determined with overrepresentation analysis using Entrez gene ID annotations. Next, a Gene Ontology (GO) enrichment analysis was performed. We focused on biologic processes and molecular functions present in the so-called GO Fat set created by DAVID. This set attempts to filter the broadest terms so that they do not overshadow the more specific terms, which is a common problem in GO enrichment analysis. Since there is no measure for the specificity of each GO term, the term specificity is based on the number of child terms, which enables filtering of the broadest terms in the hierarchy. Further analysis of differentially regulated pathways in diet groups was performed in PathVisio using KEGG pathways and WikiPathways [40,41] and DAVID using KEGG pathways.

The results of the gene expression analyses are presented in tables of gene set enrichment analyses and as tables of pathways possibly affected by diet (z-score >2) with approaches (limits in p values and fold changes) that are defined in the tables and in the Results. For clarity and the availability of gene expression analysis tools, the Entrez gene ID annotated data are presented unless stated otherwise.

### Statistical analysis

Due to the small sample size, we devised an ad hoc statistical test in the microarray analyses to identify genes of interest. The statistical significance of gene expression changes was estimated by calculating the ratio of the between-treatment and the within-treatment variance for each gene. The bead intensity variance of each gene for each treatment group, as returned by BeadStudio (Illumina Inc.), was used as an estimate of the within-treatment variance. The resulting ratio was used as an F-test statistic with the degrees of freedom set to the average number of beads used for each gene. P-values were calculated and Bonferroni’s correction method used to reduce the number of false positives. Due to the pooled nature of the microarray data, this rough ad hoc approach was chosen to increase the identification of differentially expressed genes between different diet groups rather than depending solely on fold changes.

The statistical significance of phenotypic parameters was tested between the high-fat or normal control diet groups combined and the bilberries or no bilberries groups combined using the Mann–Whitney U-test, and significantly different parameters were further tested between each diet to their corresponding control followed with Bonferroni’s correction for multiple testing in SPSS (v.17.0, SPSS Inc., Chicago, IL). The differences between the compared groups were considered statistically significant when p-values were less than 0.05.

## Results

**Figure 1:**
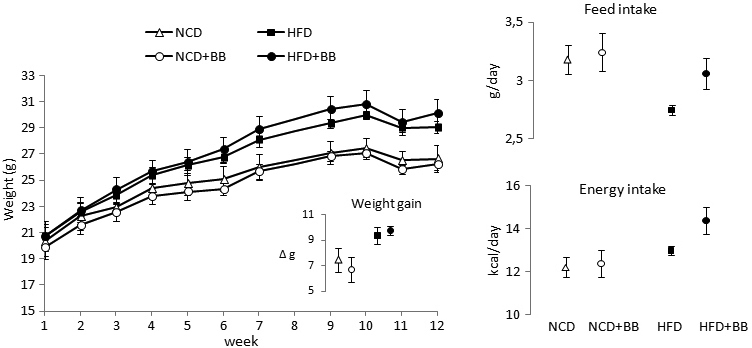
Mean cumulative weight gain, feed and energy intakes of mice in the feeding trial. C57BL/6J mice were fed for 12 weeks with normal control diet (NCD), high-fat diet (HFD), or 5% bilberries (BB) supplemented in these diets. The results are expressed as mean±standard error of the mean (SEM), n=6 mice per group housed pairs.

The effect of an HFD on phenotypic parameters was first evaluated to examine the efficacy of the mouse model. When all mice (n=24) were grouped according to the fat content of the diets (HFD and HFD+BB versus NCD and NCD+BB), there were significant differences in weight between these combined groups from week 5 and in several other parameters at the end of the study (Figure 1, Table 2, Table 3). The mice on the HFD with or without bilberries had significantly increased energy gain (p=0.011), final weight (p=0.004), and weight gain (p=0.005), elevated levels of blood glucose (p=0.018) and serum FFAs (p=0.027), as well as increased systolic blood pressure (p=0.045). The heart rates were similar in all groups (unpublished data), indicating that the animals were well habituated to the measurements. When the differences in the individual diet groups were tested, the phenotypic parameters between mice fed the HFD and the NCD did not reach statistical significance, though the HFD tended to increase the mean weight gain (+1.8 g, +25%), blood glucose (+3.5 mmol/l, +39%), FFAs (+0.2 mmol/l, +20%), and systolic blood pressure (+17 mmHg, +18%).

**Table 2:** Intake of water, feed and energy and five day weight gain of C57BL/6J mice fed with normal control diet (NCD), high-fat diet (HFD) or these diets supplemented with with 5% bilberries (BB).

**Diet**	Water*	**Feed**	Energy*	**Weight gain**
NCD	4.0±0.4	3.3±0.1	12.6±0.3	0.6±0.2
NCD+BB	3.3±0.1	3.4±0.1	12.5±0.4	0.6±0.1
HFD	4.0±0.2	2.9±0.0	13.7±0.2	0.6±0.1
HFD+BB	3.2±0.2	3.0±0.0	13.9±0.8	0.8±0.1

Water, feed (g/day/mouse) and energy intake (kcal/day/mouse) and five day weight gain (g) in the middle of the study (week 6). Data presented as mean ± SEM, n=6 mice per group housed in pairs. *Water, feed and energy intakes of 3 cages, 2 mice per cage converted to mean values per mice ± SEM.

**Table 3:** Systolic blood pressure (SPB, mmHg), blood glucose and serum free fatty acid (FFA) concentrations (mmol/l) of C57BL/6J mice fed with normal control diet (NCD), high-fat diet (HFD) or these diets supplemented with 5% bilberries (BB).

**Diet**	**SBP, mmHg**	**Blood glucose, mmol/l**	**FFA, mmol/l**
NCD	96±5 (2)	9.0±1.0	1.0±0.08 (4)
NCD+BB	97±4 (4)	9.7±1.7	1.0±0.10 (5)
HFD	113±5 (5)	12.5±1.1	1.2±0.15 (5)
HFD+BB	101±3 (2)	14.3±2.2	1.3±0.12 (6)

Results are mean ±SEM, n=6 mice per group (unless in brackets).

When grouped according to the presence of BB in the diet, the mice fed with bilberries (NCD+BB and HFD+BB) consumed significantly less water (p=0.007) than mice not fed with bilberries (NCD and HFD) in the middle phase of the study (Table 2). However, no differences were observed in the average feed intake, energy intake, or weight gain in the middle (week 6) or during the 12 weeks of feeding compared to mice not fed with bilberries (Figure 1, Table 2). The bilberry diets (NCD+BB and HFD+BB) contained 0.92 and 0.83 mg/g anthocyanins and consequently provided 2.94 and 2.66 mg/d of anthocyanins, respectively.

Bilberries in the diet had no significant effects in phenotypic parameters but tended to lower blood pressure (−12 mmHg, −11%) and increase FFAs (+0.1 mmol/l, +8%) and blood glucose levels (+1.8 mmol/l, +14%) in mice fed with the HFD (Table 3). The differences were not statistically significant, possibly due to the low number of animals in this study. Similar observations were found in the subgroups of mice (n=4 per group) used for microarrays of retinal gene expression (Appendix 2).

### Microarray transcriptomics

**Figure 2:**
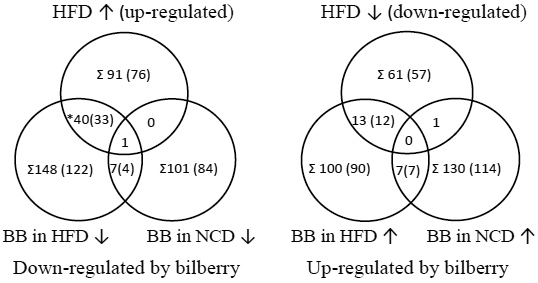
Differentially regulated transcript counts in the mouse retina after the feeding trial. C57BL/6J mice fed with normal control diet (NCD), high-fat diet (HFD) and 5% bilberries (BB) in these diets for 12 weeks. Counts from HFD up- and downregulated probes (genes) with opposite ≥1.3 fold changes (a cut-off value equal to 0.4 in the log ratio) when compared to bilberry regulated probes - HFD+BB versus HFD or NCD+BB versus NCD (Σ, the sum and gene count in brackets, * Includes the group of crystallins).

A total of 810 differentially regulated genes were identified at p<0.05 after an initial overview of the biologic processes affected (Appendix 3). These 810 genes were used in the GSE and pathway analyses. The gene expression changes in the retina were examined by comparing 1) HFD-induced expression (HFD/NCD), 2) bilberry-induced expression in obesity (HFD+BB/NCD), and 3) bilberry-induced expression compared to their corresponding controls (HFD+BB/HFD and NCD+BB/NCD). In addition, we compared the genes up- and downregulated by the HFD to genes expressed in the opposite direction by BB, parallel in both BB diets (Figure 2).

### High-fat diet induced gene expression changes in the retina

The most significantly regulated pathway found between the HFD and the NCD, within the similar number of genes up- and downregulated, was systemic lupus erythematosus (SLE) with 15 genes within the search criteria (Table 4 and Table 5). Interestingly, T2D, Gap junction, and long-term potentiation (LTP) as well as cell adhesion molecules (CAMs) pathways were significant, but with a fairly low gene number (Table 4). When analyzing either up- or downregulated genes between these diets, the mitogen-activated protein kinase (MAPK; up) and apoptosis (down) pathways displayed the highest number of differentially regulated genes (Table 5). Among the most differentially expressed genes were the crystallins (Table 6). Several crystallins such as crystallin alpha A, crystallins beta A1, A2, A4, and beta B1, B2, B3, and crystallins gamma B, C, and S showed higher expression in samples from the HFD-fed versus the NCD-fed mice, suggesting an upregulation of these genes induced by the HFD.

**Table 4:** Pathway analyses of retinal gene expression in C57BL mice fed normal control diet (NCD), high fat diet (HFD) or bilberries (BB) in these diets for 12 weeks.

**Description of group**	**Pathway**	**Genes^1^**	**Z-score^2^**
HDF vs NCD	Systemic lupus erythematosus	15	11.84
	Type II diabetes mellitus	3	3,87
	Allograft rejection	3	3,50
	Graft-versus-host disease	3	3.5
	Type I diabetes mellitus	3	3.24
	Amoebiasis	4	2.91
	Antigen processing and presentation	3	2.81
	Apoptosis	3	2,44
	Cell adhesion molecules (CAMs)	4	2,40
HFD+BB vs NCD	Systemic lupus erythematosus	14	10.85
	Protein processing in ER	5	3.06
	MAPK signaling pathway	6	2.38
	Wnt signaling pathway	4	2.19
NCD+BB vs NCD	Systemic lupus erythematosus	14	9.62
	Dilated cardiomyopathy	3	2.1
HFD+BB vs HFD	Systemic lupus erythematosus	16	11.4
	Tyrosine metabolism	3	3.99

Pathway analyses in Pathvisio using p-values and fold changes (FC) in KEGG pathways to identify the differences in the diet groups. ^1^The number of genes passed criteria of p<0.05 AND LR> 0.4 OR LR<-0.4 (equal to fold changes: FC >1.3 OR FC<1.3). ^2^The standard statistical test (z-test) under the hypergeometric distribution.

**Table 5:** Gene set enrichment analyses (GSEA) of retinal expression in C57BL mice fed normal control diet (NCD), high fat diet (HFD) or bilberries (BB) in these diets for 12 weeks.

**Limit^1^**	**Term**	**Count**	**Fold enrichment**	**p-value^2^**
**HFD versus NCD**				
**upregulated (n=431, 2 unknown)**				
	Dilated cardiomyopathy	7	3.5	0.01
	Hypertrophic cardiomyopathy (HCM)	6	3.3	0.03
	MAPK signaling pathway	11	1.9	0.06
**down regulated (n=374, 3 unkwown)**				
	Apoptosis	11	6.2	0
	Calcium signaling pathway	10	2.6	0.02
	Melanogenesis	6	2.9	0.05
	Long-term potentiation	5	3.5	0.05
**HFD+BB versus NCD**				
**upregulated (n=395, 2 unknown)**				
	Systemic lupus erythematosus	7	3.5	0.01
	Colorectal cancer	6	3.6	0.02
**down regulated (n=410, 3 unknown)**				
	Apoptosis	10	5	0
	Calcium signaling pathway	10	2.3	0.03
	Gap junction	6	3.2	0.05
**HFD+BB versus HFD:**				
**upregulated (n=393, 1 unknown)**				
	Systemic lupus erythematosus	7	3.4	0.02
**down regulated (n=412, 4 unknown)**				
	Apoptosis	9	4.7	0
**NCD+BB versus NCD:**				
**upregulated (n=416, 2 unknown)**				
	Systemic lupus erythematosus	8	3.6	0.01
**down regulated (n=389, 3 unknown)**				
	Apoptosis	9	5.1	0
	MAPK signaling pathwway	11	2	0.04
	Calcium signaling pathway	9	2.3	0.04

The gene expression enrichment results for KEGG pathways determined by over-representation analysis in DAVID.^1^ limits in search criteria up: p<0.05 AND LR> 0 OR down: LR<0 regulated probes. Log ratio (LR) determined by subtracting the denominator groups from numerator groups’ natural log-expression values.  ^2^ p values from F-test statistics with Bonferroni’s correction for multiple testing (DAVID).

**Table 6:** Differentially regulated gene expression of the group of crystallins between NCD, HFD and bilberries (BB) in these diets to NCD and HFD+BB to HFD after 12 weeks (n=13, number (#) of order in genes with p<0.01 in Supplement 4, average log ratio from 4 samples per group).

		**HFD**	**HFD+BB**	**NCD+BB**	**HFD+BB**		
**#**	**Symbol**	**NCD**	**NCD**	**NCD**	**HFD**	**Name**	**Entrez_ID**
1	*Crybb2*	3.16	1.16	0.94	−2	Crystallin, beta B2	12961
2	*Crygs*	3.08	1.16	0.72	−1.93	Crystallin, gamma S	12970
3	*Cryba2*	3.03	0.9	0.7	−2.13	Crystallin, beta A2	12958
4	*Cryba1*	2.96	0.83	0.74	−2.13	Crystallin, beta A1	12957
5	*Cryba4*	2.05	0.35	0.25	−1.7	Crystallin, beta A4	12959
6	*Cryba1*	2.53	0.76	0.68	−1.77	Crystallin, beta A1	12957
8	*Cryaa*	1.74	0.45	0	−1.29	Crystallin, alpha A	12954
16	*Crybb1*	0.77	0.1	0.12	−0.67	Crystallin, beta B1	12960
18	*Crybb3*	0.86	0.1	0.03	−0.76	Crystallin, beta B3	12962
19	*Crygb*	0.82	0.2	0	−0.62	Crystallin, gamma B	12965
28	*Crygc*	0.64	0.2	0.13	−0.44	Crystallin, gamma C	12966
55	*Cryab*	0.83	0.52	0.09	−0.32	Crystallin, alpha B	12955
69	*Crygd*	0.55	0.03	0.16	−0.52	Crystallin, gamma D	12967

### Bilberry induced gene expression changes in the retina

Genes associated with SLE, protein processing in the endoplasmic reticulum, and the MAPK pathway appear to be significantly regulated in response to HFD+BB. The SLE pathway was affected when diets with bilberries were compared to both control diets (Table 4 and Table 5). The number of significantly regulated genes within the SLE pathway (14–16 genes) remained similar in all groups. In addition to the previous pathways, downregulated genes in retinas of mice fed with bilberries compared to both control diets displayed significant regulation of the apoptosis pathway. The MAPK pathway was affected in the HFD+BB group (Table 4) and displayed potential downregulation in NCD+BB (Table 5). This may indicate that bilberries are able to induce differential regulation of genes within the MAPK pathway. If one examines the most differentially expressed (p<0.01, n=131) genes between all groups, then a total of 13 were crystallins (beta, gamma, and alpha) with mitigated expression in the HFD+BB group (Table 6, Appendix 4). These crystallins were furthermore potentially downregulated in HFD+BB to the HFD.

### Bilberry induced gene expression changes in high-fat-diet upregulated genes in the retina

Further gene enrichment approaches were applied to clarify the significance of either HFD versus NCD up- or downregulated genes having opposite fold changes in the groups fed bilberries. This was performed to explore molecular functions or biologic processes in the absence of differently regulated pathways. The results revealed that apoptosis and MAPK and the biologic process of eye development and molecular function of the structural constituents of the eye lens seemed to be modulated by bilberries. HFD up- (359, 76) or HFD down- (318, 57) regulated genes with opposite fold changes in each group with bilberries are described with the number of identifiers recognized in DAVID (0, 1.3 fold change [FC] for cut-off value). A zero cut-off was used since with FC≥1.3 the number of genes with opposite FC was too low for GSEA (Figure 2). While simultaneously upregulated by HFD and downregulated by HFD+BB versus HFD (250, 33), the enrichment of genes displayed no previously shown pathways but pointed to the molecular functions of the structural constituents of the eye lens as being significant (13 genes, p=4.9E-18, 46.7 fold enriched), including 12 crystallins (Table 2) and the major intrinsic protein of eye lens fiber (*Mip*). These genes were also found within the biologic process of eye development (12 genes, p=4.4E-6, 6.1 fold enriched). These molecular functions and biologic processes were furthermore found in HFD upregulated genes when compared to genes downregulated by HFD+BB versus NCD (161, 77). Altogether, the HFD+BB group showed similar genes (crystallins) differentially regulated compared to either background diet, and six of these genes were expressed with ≥2.5 FC in the HFD (Figure 2, Table 6).

### Bilberry induced gene expression changes in high-fat-diet downregulated genes in the retina

If one examines the HFD downregulated genes that were upregulated in HFD+BB versus HFD (222, 12), the apoptosis pathway (four genes, p=0.081, 3.9 fold enrichment) was shown with a probe for tumor necrosis factor-alpha (TNF-α) also recognized in the SLE pathway. HFD downregulated and HFD+BB versus NCD upregulated only a low number of genes (128, 0) that were enriched within two pathways: colorectal cancer (four genes, p=0.022, 6.5 fold enriched) and melanogenesis (four genes, p=0.032, 5.6 fold enriched). The apoptosis pathway (4 genes, p=0.021, 6.6 fold enriched) was enriched with HFD down- and BB in NCD upregulated genes to NCD (123, 1), but surprisingly it was also enriched within the downregulated genes in the mouse retina of NCD+BB to NCD (Table 5) indicating that NCD+BB may influence multiple genes via increased and decreased expression in this pathway.

### Bilberry induced gene expression

BB in both diets (NCD+BB to NCD and HFD+BB to HFD) increased the expression of 204 genes enriched in the SLE pathway (histone clusters 1 *H2aa*, *H2ad*, *H2af*, and *H4k* and histone cluster 2 *H2ac*; p=0.016, 5.1 fold enrichment) and the glutathione metabolism pathway (glutamate-cysteine ligase [*Gclm*], glutathione S-transferase theta 2 [*Gstt2*], glutathione peroxidase 5 [*Gpx5*], thioredoxin domain-containing 12 [*Txndc12*], p=0.013, 8.0 fold enrichment). The genes (197) downregulated by both BB diets were found enriched in the apoptosis (caspase 8 [*Casp8*] and fas-associated protein with death domain (FADD)-like apoptosis regulator [*Cflar*], cyclic adenosine monophosphate (cAMP) dependent protein kinase catalytic alpha and catalytic beta [*Prkaca, Prkacb*] and protein phosphatase 3, catalytic subunit, isoform gamma [*Ppp3cc*], p=0.001, 5.8 fold enrichment) and MAPK (*Prkaca*, *Prkacb*, *Ppp3cc*, dual specificity phosphatase 16 and 18 [*Dusp16, Dusp8*], serine/threonine kinase 4 [*Stk4*], voltage-dependent calcium channel beta 4 subunit, p=0.043, 2.7 fold enrichment) pathways. Moreover, these pathways were also discovered among the genes (105) downregulated by HFD and upregulated by BB (glutathione metabolism: *Gpx5*, *Gstt2*, *Gclm*; p=0.032; 10.3 fold enriched), and among the genes (120) upregulated by HFD and downregulated by BB (MAPK: *Stk4*, *Prkaca*, *Prkacb*, *Dusp8*, *Dusp16*, p=0.07, 3.1 fold enrichment). These consistent changes in gene expression, induced by BB in opposite direction to the HFD-regulated genes, clearly point to diet-dependent transcriptional regulation and furthermore were not attributable to the control diets.

## Discussion

Obesity and diabetes are well recognized risk factors for ocular diseases. Several disease models in rodents have been successfully created to study obesity-associated phenotypic changes at the tissue level. The most commonly applied models of diabetes involve STZ treatment that evoke compromised glycemic homeostasis and thus represent a model of type 1 diabetes resulting from decreased insulin secretion due to gradual pancreatic beta-cell death. By using STZ in rodents as well as genetic models, numerous advances have been made in the pathology of diabetes and age-related progression of retinal disorders. However, only a few studies have used C57BL mice with an HFD to evaluate the retinal gene expression changes related to the gradual progression of diabetes. In this study, we used the microarray screening approach and identified similar changes in crystallin expression as found in the previous models using STZ. We also show how bilberries rich in anthocyanins, known for their anti-inflammatory and antioxidative properties, seem to counteract the major gene expression changes found in mice retinas due to consumption of an HFD. The differences in phenotype parameters and in the expression of transcripts in mice retinas between the HFD and the NCD with or without bilberries will be discussed here to explore the possible effects of bilberries at the molecular level.

### Phenotype

The HFD- and HFD+BB-fed mice exhibited increased weight from 5 weeks of the trial and had elevated weight gain and systolic blood pressure as well as higher FFA and non-fasting blood glucose concentrations. Due to the small number of mice and the high variation, statistically significant differences were only partly observed between the HFD and NCD groups after correction for multiple testing. Nevertheless, the mice fed the HFD in the present study can be regarded as being in a prediabetic state. The mice fed HFD+BB displayed increased weight gain and reduced systolic blood pressure at the end of the study; these findings were similar to those previously reported [24,42]. Increased weight gain could result from the improved peripheral insulin sensitivity reported after treatment with anthocyanins [43] or consumption of blueberries [44], since increased insulin sensitivity is known to lead to increased energy storage. In conclusion, the possible changes in retinal gene expression induced by bilberries cannot be attributed to blood glucose levels or other phenotypic differences between groups since these parameters were apparently not affected by the inclusion of bilberries in the diet.

### Retinal gene expression

The retinal gene expression studies in diabetic rats and mice revealed changes in inflammatory, antivascular barrier, and neurodegenerative genes associated with increased apoptosis and vascular permeability [9,10,12,45,46]. The previous studies used either STZ triggered type 1 diabetes (T1D) in mice and rats or the genetic T1D mouse model strain, Ins2^Akita^ [47]. Our results provide additional screening data since mice fed an HFD generally display gradual changes in inflammation, glucose, and lipid metabolism leading to T2D. We found differential expression of genes in the SLE, apoptosis, and MAPK pathways and particularly in the transcripts for crystallins. Overall, the gene expression changes due to diets were evenly distributed between up- and downregulated genes as previously described [12]. In a microarray experiment (12 weeks), strain C57BL/6J mouse models of STZ and Ins2^Akita^ mice (GSE19122) induced changes in 333 and 404 probes related to 307 and 392 differentially expressed genes evenly distributed between up- and downregulated genes in these two diabetes models, respectively (p<0.05 and >1.4 FC). Despite finding fewer changes than in the other diabetic mouse models, our results were also evenly distributed with a total of 91 (76) HFD upregulated and 61 (57) downregulated probes (and genes) within the 810 (683) differentially expressed transcripts (p<0.05, ≥1.3 FC). This can be explained by the statistical approach and the use of pooled samples, and thus there is less statistical power than in previous studies.

### High-fat diet induced gene expression

In our study, crystallins displayed the highest increase in HFD-associated gene expression. Crystallins were initially isolated as structural proteins in the lens, but their expression has been observed in the mouse retina [48]. Alpha crystallins are molecular chaperones, acting as regulators of apoptosis and vascularization in the retina, and they are closely involved in neuronal inflammation and oxidative stress resistance [49]. Whereas the alpha crystallins are recognized as small heat shock proteins, beta and gamma crystallins are less known for other than their structural role in the lens. Nevertheless, beta and gamma crystallins have also shown increased gene expression in animal models of retinal diseases or stressed conditions, for example, diabetic retinopathy and light-induced damage [50,51]. Not only differently regulated crystallin transcripts but also the actual proteins have been identified in retinas of diabetic mice, i.e., the Ins2^Akita^ strain and in the C57BL strain after STZ treatment [13], as well as in rats [52,53]. Furthermore, increased levels of alpha-crystallins have been determined in diabetic humans [54]. The increased subtypes, i.e., alpha, beta, and gamma crystallins observed in diabetes, are expressed in glia cells (alpha) and neurons (beta, gamma) [49]. In diabetes, the antiapoptotic functions of alpha-crystallins in the retina have been shown to be disrupted, leading to controlled neuronal cell death [13]. In our study, the HFD increased the expression of crystallins beta A1 A2, A4, and B2 and crystallins alpha A and gamma S. We also found enrichment of the apoptotic pathways, which were among the genes affected by the HFD, to further support the proposal that the HFD model may possess similar gene expression characteristics in rodents as in STZ-induced or Ins2^Akita^ mice exhibiting the diabetic phenotype.

Within the HFD-induced pathways such as T2D, Gap junctions and CAMs have also been identified in previous studies with other mouse models. Gap junctions are involved in neuronal coupling of cones, rods, and amacrine cells in the retina [55], and tight junctions are associated with CAMs in the blood–retinal barrier (BRB) of endothelial cells. The breakdown of the BRB following vascular leakage has been proposed to be involved in the pathogeneses of diabetic retinopathy, ischemic retinopathies, and macular edema. The BRB breakdown is thought to consist of two alternative pathways, one involving endothelial cell tight junctions and the other mediating vehicle transport by caveolae. The altered gene expression evoked by STZ pointed to transient changes in paracellular and prolonged disruption of vesicular transportation causing the increased permeability found in retinal capillaries in preclinical diabetic retinopathy [10]. Furthermore, the BRB breakdown in diabetic retinopathy has been shown to require TNF-α [56]. A previous study using mice fed an HFD (21% fat, 12 weeks) observed increased expression of *Tnf-α*, and a modest increase in the expression of P-selectin and E-selectin [46]. If one considers the genes involved in the BRB, *Lgals3bp* has been a common gene upregulated in two mouse models of diabetes [12], and was potentially upregulated in this study. *Tnf-α* has been found as a common gene in several HFD affected pathways but was not upregulated in this data set. Nevertheless, one must be cautious in interpreting dietary effects on individual genes without applying other molecular approaches. These results merely indicate a possible change in these pathways and the average change in genes within these pathways.

The most predominant pathways observed in all groups were the SLE and MAPK pathways, although found within the HFD upregulated transcripts. These both indicate that innate and adaptive immune responses were affected by the HFD. Previous studies have detected changes in retinal gene expression related to inflammation in diabetic models [12,46]. An HFD has increased the expression of *Tnf-α*, intercellular adhesion molecule 1 (*Icam-1*), interleukin-6, and interleukin-1-beta in the retina [46], and network analyses of two mouse models centered on p38MAPK (Ins2^Akita^) and T cell receptor (TCR), interferon (IF)-alpha, and beta (STZ) in mice [12]. Parainflammation has been described in the progression of AMD [57], diabetic retinopathy [58,59], and obesity [60], and the transcripts in aging and diabetic retinas have been shown to share commonalities [45].

### Bilberries potentially modify retinal gene expression

Genes associated with SLE, protein processing in the endoplasmic reticulum, and MAPK pathways were found in the pathways affected by HFD+BB, and SLE with respect to NCD+BB. *Tnf-α*, a commonly observed transcript in the previous pathways, and vascular endothelial growth factor, a highly relevant transcript according to previous studies, were both downregulated in the BB diets. However, the most enriched and interesting gene group, while acknowledging the study design limits, was the significant downregulation of crystallins in the BB in HFD-fed mice to HFD. As described in the HFD-induced transcripts, especially alpha crystallins have been detected in rodents [13,49,52,53] as well as in post-mortem retinal samples of diabetic patients [54]. Furthermore, these proteins have been shown to have a functional role in regulating apoptosis in diabetic mice [13]. Moreover, differently regulated pathways of HFD-induced MAPK signaling and apoptotic pathways were both found to be enriched in downregulated genes from animals on the BB diets. The results may be evidence of the dampening of the expression of genes involved with these pathways. The MAPK and SLE pathways were induced by bilberries commonly in the opposite direction to HFD alone, but in contrast to the mechanisms involving oxidative stress, they have not been elucidated before in the retina in such detail. Nevertheless, extracts from bilberries have displayed anti-inflammatory properties and been able to protect photoreceptor cell function in a mouse in vivo model of endotoxin-induced uveitis [30]. Therefore, further clarification of these pathways would be clearly beneficial.

However, the most convincing commonly upregulated pathway by the BB diets was the glutathione metabolic pathway. Upregulation of this pathway is in accordance to previous in vitro and in vivo findings. The bilberry anthocyanidins (cyanidin, delphinidin, and malvidin) have well known neuroprotective properties in mouse retinal ganglion cells in vitro and in vivo [61], in vitro human RPE cells [62], and in rabbit models [63] in combating reactive oxygen species–induced damage. Even short-term exposure to anthocyanin or non-anthocyanin phenolic fractions of bilberry extract has increased the activities of oxidative stress defense enzymes of heme-oxygenase (HO)-1 and glutathione S-transferase-pi (*Gst-pi*) in human RPE cells [64]. Bilberry extracts have also increased glutathione levels and the gene expression and activities of total superoxide dismutase and glutathione peroxidase (*Gpx*) in mice [29–31]. In the present study, the genes for *Gst* and *Gpx* were found within the BB-upregulated and HFD-downregulated pathway of glutathione metabolism. Therefore, bilberries may indeed possess the activity to induce the antioxidant defense pathways.

As far as we are aware, the current study represents the first screening of short-term obesity-related changes in the retina using a high-fat diet, i.e., 45% energy from fat. Although the mouse model used in this study is well characterized and uses an open source diet, two additional issues in gene expression analyses have to be considered before drawing any final conclusions. The retinal isolates are multicellular samples representing the average expressional difference of genes from several cell types in the intact retina, and second, the microarray samples are pooled and represent the average gene expression from four mice. Nonetheless, our data point to a similar progression of molecular events as have been described with other mouse models of diabetes and obesity. We have shown that bilberries apparently counteract the increase in crystallin expression evoked by an HFD. Other bilberry-specific changes may include inducing genes in the oxidative stress–related pathway, e.g., glutathione metabolism, and they may be responsible for the antioxidant properties of these berries. In addition, dampening of the apoptosis and MAPK-related pathways may be beneficial in preserving ocular health. These findings still need to be further verified and explored in protein levels but clearly demonstrate the potential efficacy and molecular mechanisms of bilberries for maintaining the health of the eye. Thus, bilberries represent a highly promising source of compounds for protecting the retina from the harmful, inflammatory, and oxidative stress–related effects of diets rich in saturated fat.

## Appendix 1. Diet composition.

Manufacturer: Research Diets, Inc. Twenty Jules Lane, New Brunswick, NJ 08901. To access the data, click or select the words “Appendix 1.” This will initiate the download of a compressed (pdf) archive that contains the file.

## Appendix 2.

Systolic blood pressure (SBP, mmHg), glucose and serum free fatty acid concentrations (mmol/l) from subgroups of mice used in retinal gene expression analyses after 12 week ad libitum feeding on normal control diet (NCD), high-fat diet (HFD) or 5% (w/w) freeze-dried bilberries (BB) in either diet (Mean±SEM, 2–4 per group with the number of animals in brackets). To access the data, click or select the words “Appendix 2.” This will initiate the download of a compressed (pdf) archive that contains the file.

## Appendix 3. Gene Ontology (GO) enrichment analysis on the subset of biologic processes present in GO.

Fat set created by DAVID (n=810 probes) from differently regulated genes expressed in pooled mouse C57BL/6J retina between all groups; normal control (NCD), high-fat diet (HFD), normal or high-fat diet with bilberries (BB) fed ad libitum for 12 weeks. To access the data, click or select the words “Appendix 3.” This will initiate the download of a compressed (pdf) archive that contains the file.

## Appendix 4.

Differentially regulated genes in mouse retina between NCD, HFD and bilberries (BB) in either diet to NCD after 12 weeks (n=131, F-test, p<0.01). To access the data, click or select the words “Appendix 4.” This will initiate the download of a compressed (pdf) archive that contains the file.
